# Possible Perspectives of P6 Acupressure

**DOI:** 10.5812/nms.10507

**Published:** 2013-06-27

**Authors:** Alessandro Micarelli, Andrea Viziano, Isabella Pavone, Marco Alessandrini

**Affiliations:** 1 Department of Medical Sciences and Translational Medicine, Tor Vergata University, Rome, Italy

**Keywords:** P6 Acupressure, Perspectives, Possible

Dear Editor,

We read with great interest the article by Etri M et al. about the effects of P6 acupressure on pain and vital signs of patients following small abdominal surgeries (1). Post-operative pain is often experienced by surgical patients, with practical consequences on clinical outcomes, due to the possible complications related to the use of analgesic drugs (such as opioids or non-steroidal anti-inflammatory drugs). Moreover, the experience of discomfort complained by the patients may affect their subjective judgment about surgical procedures. For these reasons, there is a large amount of studies among the existing literature about the effects of alternative medicine - especially the acupressure - in the management of post-operative pain and other common symptoms such as nausea, vomiting, insomnia, muscular pain and dyspnea. However, there are still not univocal evidences supporting the routinely use of such procedures. Possible explanations about this lack of evidences may be the small amount of randomized controlled trials which have reached an adequate sample size as well as the differences in acupressure dose administration between the studies (2).

Etri et al. study (1) investigated the effects of P6 acupressure on vital signs in post-operative patients and a possible relationship between acupressure and a more objective measure of its efficacy was suggested. We think that the purpose of verifying the effects of an alternative medicine in less subjective way than individual symptoms is a valid and interesting choice. The method adopted for the study consisted in randomizing the two groups of patients with respect to some variables, such as age, duration of anesthesia and length of surgical incision. Moreover the double-blind effect (patients did not know which group they were belonging to) added scientific value to the study.

Analysis of vital signs is crucial in the post-operative period; nevertheless any evidence has been found at this moment about a possible relationship between P6 acupressure and post-operative changes of systolic and diastolic blood pressure, respiration and pulse rate, both in the general literature and in the aforementioned study (1). This may suggest that P6 acupressure has no role in blood pressure, pulse rate and respiratory activity alteration in post-operative phases. Eventually, different way of investigation or different study design need to be encouraged in order to explain P6 related vital signs changes. 

With respect to the results of the study about post-operative pain, no statistically significant effect of P6 acupressure was found in any of the seven measurements, and patient-assessed pain severity scores were essentially the same in the P6 and Control groups. The mean of pain severity was even higher in the P6 group in two occasions. In this field, studies about pain management by using acupressure showed ambiguous results: effects of P6 Acupressure on post-surgical pain were not frequently analysed, while the majority of scientific works concerning acupressure and pain focused on other topics such as dysmenorrhoea, labour pain, back and neck pain and minor trauma. Overall, the global results suggested, with a good level of evidence, a positive effect of acupressure (both in the P6 point and in other points) on these forms of pain (3).

With respect to what mentioned above and in the existing literature we believe that P6 acupressure might be considered in the management of several conditions. For example, in our experience (4) we evaluated the effectiveness of P6 acupressure on vertigo, neurovegetative symptoms and its clinical usefulness during acute vestibular disorders. The study was designed as a randomized clinical trial (RCT) with patients divided in an experimental and in a placebo group, the latter with the P6 band bilaterally placed in an inappropriate way. Patients of both groups were asked to subjectively evaluate the severity of vertigo and neurovegetative symptoms on a visuo-analogue scale ranging from 0 to 10, before and after the bilateral placement of P6 device for 30 minutes. The device demonstrated to be effective in ameliorating symptoms, especially neurovegetative ones (i.e. nausea and vomiting), in patients with spontaneous and provoked vertigo. Moreover P6 acupressure demonstrated not to interfere with vestibular-ocular reflex (VOR) and thus not to reduce the reliability of vestibular tests. This topic has to be further highlighted considering that the literature is univocal in recognising the VOR suppressing effect of common anti-vertiginous drugs (4).

Our study findings suggest that P6 acupressure could be a valid alternative to anti-vertiginous and anti-emetic drugs in the management of neurovegetative symptoms related to acute vertigo: this could be explained by its peculiar effect in modulating the neural centers responsible for nausea and emesis genesis. In fact, P6 acupressure has been proven to prolong slow waves of gastric peristalsis and to reduce antiperistalsis by stimulating the electrical discharge in the dorsal motor nucleus of the vagus nerve (5). Other biological effects of P6 acupressure, or related to the electrical stimulation of the same region, are still under investigation and more researches are required. However, in the current literature few acupressure RCTs have reached an adequate number of patients and the placebo effects biasing this technique are still difficult to quantify. Thus, we think that further studies on this topic are strongly needed, especially in the field of pain management.

We are confident that with more trials, such as the study proposed by Etri M and co-workers, the effective clinical usefulness of P6 acupressure could be defined. This may be helpful to physicians and nurses, because it could be a safe and low cost approach in order to relieve the patients from unpleasant symptoms.
